# Principles and Clinical Uses of Real-Time Ultrasonography in Female Swine Reproduction

**DOI:** 10.3390/ani9110950

**Published:** 2019-11-11

**Authors:** Johannes Kauffold, Olli Peltoniemi, Axel Wehrend, Gary C. Althouse

**Affiliations:** 1 Clinic for Ruminants and Swine, Faculty of Veterinary Medicine, University of Leipzig, An den Tierkliniken 29, 04103 Leipzig, Germany; 2 Department of Production Animal Medicine, Faculty of Veterinary Medicine, University of Helsinki, Paroninkuja 20, 04920 Saarentaus, Finland; olli.peltoniemi@helsinki.fi; 3 Clinic for Obstetrics, Gynecology and Andrology of Large and Small Animals, Faculty of Veterinary Medicine, Justus-Liebig-University, Frankfurter Strasse 106, 35392 Giessen, Germany; Axel.Wehrend@vetmed.uni-giessen.de; 4 New Bolton Center, School of Veterinary Medicine, University of Pennsylvania, 382 West Street Road, Kennett Square, PA 19348, USA; gca@vet.upenn.edu

**Keywords:** swine, reproduction, ultrasonography, imaging, diagnostics

## Abstract

**Simply Summary:**

Real-time ultrasonography (RTU) has become an essential diagnostic value when assessing female swine reproduction in either individual or groups of animals. Diagnostic application of RTU is applied throughout most stages of production, including gilt development, breeding, gestation and farrowing. Along with its most common use in on-farm assessment of pregnancy status, RTU is also used to troubleshoot disruptions in reproductive performance such as delayed puberty, prolonged wean-to-estrus interval, absence of post-weaning estrus, decreased conception and farrowing rates, vulval discharge, peripartum and puerperal disorders. This review aims to provide an overview on principles and clinical uses of RTU in female reproduction on commercial swine farms.

**Abstract:**

Within the past 30 years, through ongoing technology and portability developments, real-time (b-mode) ultrasonography (RTU) has increasingly become a valuable diagnostic tool in assessing the female reproductive tract in swine. Initially applied in swine production to visually determine pregnancy status, RTU use has expanded to include assessment of the peri-pubertal and mature non-pregnant females as well. Transabdominal and transrectal modalities to visualizing the reproductive tract in swine have been reported with the transabdominal approach more common due to the fact of its ease of accessibility, animal/personnel safety, and reduced time to perform. Adjustable frequency transducers are preferred as they allow optimization of image quality at various depths. If a single transducer frequency must be selected, a 5 MHz probe provides the best versatility for visualizing the reproductive tract in swine. Other basic requirements for ultrasound equipment which will be used on commercial swine farms include being light weight and easy to handle, readily cleanable and disinfectable, long battery-life, and good durability. When using RTU for pregnancy determination, diagnosis is based upon a combination of the animal’s breeding records, the presence of embryonic fluid, and, depending upon gestational stage, fetal structures. If RTU is used as a diagnostic tool in assessing reproductive problems in an individual or a group of animals, sonographic evaluation of both the uterus and ovaries is performed. Tissues are delineated and assessed based upon their echogenicity, echotexture, and size. Uses of RTU in clinical practice may include assessment of delayed puberty, prolonged wean-to-estrus interval, absence of post-weaning estrus, herd disruptions in conception and farrowing rates, vulval discharge, peripartum and puerperal disorders. This review aims to provide an overview on principles and clinical uses of RTU with respect to application to address female reproductive performance issues in commercial swine operations.

## 1. Introduction

Over the past 30 years, through ongoing technology and portability developments, real-time ultrasonography (RTU) has increasingly become a valuable diagnostic tool in assessing the female reproductive tract in swine. As comprehensively reviewed twelve years ago [1], RTU has demonstrated great value in its initial application for determining pregnancy status, achieving an overall accuracy that is now superior to other determination methodologies such as behavioral assessment (e.g., return-to-estrus) or A- and Doppler-mode technologies [2,3]. Beyond pregnancy diagnosis, RTU has the ability to assess the non-gravid uteri of gilts and sows through characterization of parameters such as fluid echogenicity, echotexture, and size [1,4]. Evidence of acute/subacute endometritis, typically associated with uterine fluid accumulation with or without vulvar discharge, are ultrasonographically diagnosable [4,5]. Recent work into perfusion characteristics of the healthy uterus via RTU continues to show promise for diagnosing subtle uterine diseases such as chronic endometritis [6], and into health of the puerperal uterus during lactation [7]. 

Higher quality RTU imagery has allowed transabdominal assessment of the ovary, with most physiological and pathological ovarian structures able to be delineated [1,8]. This includes ovulatory dynamics, which is valuable in determining pubertal status of gilts and in development of timed insemination programs [1,9,10,11]. 

Collectively, RTU has become an indispensable tool for troubleshooting disruptions to production parameters such as conception and farrowing rates, problems with delayed or failed puberty attainment, and with delayed or failed post-weaning estrus issues [12,13]. This review aims to provide an overview on principles and clinical uses of RTU with respect to application in female swine in commercial operations.

## 2. Technical Requirements and Route of Application

Basic requirements for ultrasound equipment for use at swine facilities include being light weight and easy to handle (i.e., portable), easy to clean and externally disinfectable, capable of high resolution, long battery-life, and durability [1]. To optimize biosecurity, a farm should have an on-site ultrasound machine. Machine designs which avoid ventilation fans are easiest to clean and disinfect [14]. Equipment with ventilation fans is much more difficult to clean, allowing it to harbor and subsequently exhaust pathogens (i.e., PRRSV) [14]. If a machine is equipped with a ventilation fan, disposable plastic bags or cling-film may be used for covering equipment for a short period of time during use on the farm [15]. If transporting equipment between farms, be cognizant of disease status of the farms being visited. Disease status and disinfectability will dictate length of equipment downtime.

It is preferred that transducer probes be either a sector or convex (ideally electronic) configuration, as linear probes may be difficult to hold appropriately when performing transabdominal scanning. If transrectal scanning is preferred by the operator, only linear probes are appropriate. With newer generation machines, transducer probes usually provide a frequency spectrum (e.g., 3–9 MHz), with capabilities to adjust frequencies to tissue characteristics and depth in order to generate optimum images. If a single transducer frequency must be selected, a 5 MHz is preferred as lower frequencies typically provide for lower resolution insufficient for visualizing subtle structures (i.e., ovaries), while higher frequencies may lack the ability to penetrate sufficiently to visualize the entire reproductive tract.

Both transrectal and transabdominal scanning of the gilt/sow is possible [1,16]. Transrectal scanning of large sows can be performed with the probe being hand-held and manually guided. In smaller animals, the probe typically has to be attached to a stabilizing rod, usually purpose-designed plastic or metal apparatus, due to the narrow pelvis which curtails manual guidance through the rectum. The transrectal approach typically requires the prior removal of feces in order to have an adequate tissue/probe interface for capturing the best image. Transabdominal scanning is performed by placing the transducer on the lower flank above the mammary glands in the inguinal area. Transrectal versus transabdominal scanning is a matter of preference, and while transrectal scanning may facilitate a more detailed image (which is transducer quality and frequency dependent), the transabdominal approach is readily accessible and, thus, quicker to perform. The transabdominal approach also significantly reduces risk of injuries to both the animal and the person performing the technique and has the advantage of being able to be performed on both confined and loose housed sows/gilts. 

## 3. Reproductive Organs and Functions to be Monitored

Reproductive organs typically assessed using RTU are the uterus and the ovaries [1,7]. The cervix can also be imaged if necessary. Under normal circumstances, the vagina, broad ligament, and oviducts cannot be imaged using RTU. Topographically adjacent to the reproductive organs is the urinary bladder, which is readily visible [17], but will not be reviewed herein. 

### 3.1. The Uterus

Using conventional RTU, the uterus is characterized based on intrauterine fluid echogenicity (if any), echotexture, and size [1]. Echogenicity, as measured based upon grey scale analysis, may also provide for an added parameter of assessment [18]. Recent work has also been done into uterine perfusion characteristics using Doppler ultrasound technologies, but this work is still in its infancy [6]. 

#### 3.1.1. The Pregnant Uterus, Pregnancy Determination and Peripartum Assessment

Pregnancy determination has been diagnosed based on breeding records and the presence of embryonic fluid of approximately 1.0 mm in diameter as early as day 9 gestation [19]. By day 15, the conceptus measures approximately 4.0 mm and by days 18–22 is approximately 10 mm in diameter [20]. Advanced pregnancy is associated with increased accumulation of embryonic fluid and visualization of fetal tissue. When using a 7.5 MHz transducer, the conceptus is easily visualized by day 18 gestation [21] and with a 5.0 MHz transducer by 19–20 days of gestation [2,22]. Fetal heartbeats appear between days 21–25 [22,23], and are useful for the determination of fetal viability. Doppler ultrasonography via a laparoscopic approach has been used to assess umbilical arterial blood flow indices in feti at 36, 42, and 51 days of age, but the study failed to show any correlation of perfusion indices and fetal growth [24]. Visualization of fetal eye orbit and stomach occurs around day 49 of gestation (transabdominal scanning; 5.0 MHz) [22]. Although it is common to have breeding dates available in commercial operations, measurement of fetal crown–rump length can be helpful for the determination of the stage of gestation [1]. Differences in uterine echogenicity, based on grey-scale analysis, have been reported to be useful in determining a pregnant versus non-pregnant gilt at days 12–14 gestation [18]. This work hypothesized that the decreased uterine echogenicity associated with pregnancy was the result of embryo-derived estrogens eliciting an endometrial edema and hyperemia that typically occurs at the time of maternal recognition of pregnancy. 

Real-time ultrasonography can be used to diagnose pathological conditions such as embryonic death and abortion based upon the lack of ultrasonographical characteristic patterns specific to the stage of pregnancy [1,25]. Hydrometra/mucometra [1] and fetal mummification [21] using RTU have been documented as well. 

Successful diagnosis of pregnancy status in early gestation is dependent upon the machine’s quality and the skills and experience of the person performing the examination [1]. Generally, when using RTU for pregnancy determination, the challenge is to be able to perform a complete uterine scan in order to rule out pregnancy. Having assessed the use of different transducer frequencies along with their application on known days post-breeding, it is the author’s opinion that use of a 5 MHz transducer starting around day 20–21 post-breeding provides an optimal degree of accuracy in determining whether a bred animal is pregnant or is expected to return to estrus. This recommendation allows farm personnel the opportunity to focus their estrus detection pressure via boar exposure on a specific set of animals. On many commercial farms, animals which are determined during the pregnancy check as positive on initial examination have their pregnancy status re-confirmed several weeks later. While this procedure has some validity in principle, it has been found to not be necessary on farms where results from the first examination do not differ from eventual farrowing data [25].

**Figure 1:**
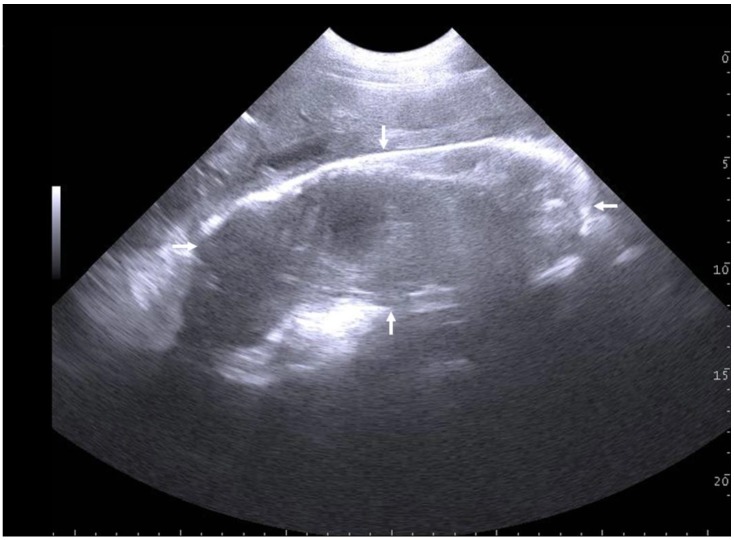
Transabdominal ultrasonographic image of a non-expelled dead piglet (arrows indicate vertical and horizontal dimensions) at examination 23 h 50 min after the beginning of farrowing. No heart beats could be detected. The sow had expelled 13 piglets and 4 placentae within 9 h 30 min, but then stopped farrowing. Post RTU and an additional vaginal examination, oxytocin was given and the retained dead piglet including the placenta expelled within 20 min. Scale bar on right margin in 1.0 cm steps. (Courtesy of Alexander Grahofer, Bern, Switzerland).

Applications of RTU peripartum have been described as well. Doppler RTU to assess fetal heart rate (FHR) antepartum is a good predictor of fetal viability [26], with serial ultrasound showing decreases to FHR within the last 10 days of gestation [27]. From an obstetrical standpoint, peripartum RTU can be useful to determine if a sow has retained piglets, along with their viability status via FHR (Figure 1) [28]. Peripartum visualization of the ovaries for the presence of non-regressed corpora lutea (CL; refer to Section 3.2. and Figure 7B) can alert farrowing personnel to a sow that may exhibit prolonged parturition and reduced colostrum production due to the presence of a higher than normal progesterone concentration [7]. Lastly, RTU can be used to correctly identify stillborn piglets through thoracic assessment of the lung tissue, where piglets born alive have ventilated lungs which ultrasonographically differ quite remarkably in echotexture from that of non-ventilated lungs [29]. 

#### 3.1.2. The Puerperal Uterus

Real-time ultrasonography has gained recent attention in the characterization of the puerperal (e.g., during lactation) uterus and uterine involution. In a study conducted in Finland, the uterus was investigated based on size (cross-sectional area of uterine horns) and intrauterine fluid between days 2 and 7 post-partum [30]. The size of the uterus decreased from 4.19 ± 1.23 cm^2^ on day 2 to 3.53 ± 0.86 cm^2^ on day 7. Sizes were also categorized as “normal” or “enlarged” based on being below or above mean values, respectively. Not surprisingly, sows with “enlarged” uteri were older parity sows, had more total and stillborn piglets, and had been exposed to more obstetrical interventions. Uterine fluid accumulation, an indicator of metritis, was also positively associated with the number of total and stillborn piglets, and animal exposure to obstetrical interventions. These results support that both “enlarged” uteri and fluid accumulation may be used as ultrasonographical parameters to ascertain puerperal disorders and acceptable uterine involution. 

**Figure 2:**
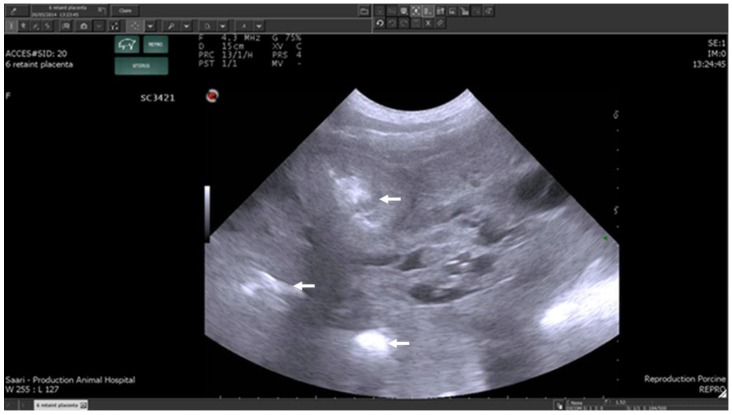
Transabdominal ultrasonographic image of cross sections of uterine horns of a sow on post-partum day 3 with retained placentae, as indicated by intrauterine hyperechogenic material (arrows). Scale bar on right margin in 1.0 cm steps [30].

Additional work has shown an association between duration of farrowing and puerperal complications [31]. Sows with a prolonged duration of farrowing (i.e., >6 h) had a higher incidence of partially or completely retained placentae (Figure 2), with complete placental retention observed in up to 6% of cases. Incidentally, administration of oxytocin after the expulsion of the last piglet was found to be beneficial in reducing the occurrence of retained placentae [31]. Along with the aforementioned puerperal events, prolonged farrowing and related uterine complications have been associated with disruption in the animal’s future fertility potential. In a study involving 148 crossbred sows, prolonged farrowing clearly increased the risk of repeat breeding in the following post-weaning cycle [32]. In another study, uterine involution in loose housed and crated sows was assessed in relation to subsequent farrowing production parameters [33]. Sows were transabdominally imaged to obtain uterine horn diameter daily between days 2 and 14 post-farrowing, and the day before weaning (e.g., 28 day lactation). Overall, both loose housed and crated sows showed similar reductions in uterine horn diameter at day 2 (loose housed: 32.4 mm; crated: 38.5 mm) and the day before weaning (loose housed: 9.0 mm; crated: 10.1 mm). Interestingly, at day 11 post-farrowing, a negative correlation was found between uterine diameter and subsequent farrowing total born and live born piglet numbers, albeit only in loose housed sows. Specifically, odds ratios for having more than 16 total born and more than 15 live born increased by 2.2 and 1.4, respectively, in animals exhibiting a uterine horn diameter of 12 mm or less compared to those with larger uterine diameters. Collectively, this body of work suggests that RTU imaging of the puerperal sow may provide added value in forecasting subsequent fertility potential.

#### 3.1.3. The Non-Gravid Post-Puerperal Uterus 

**Figure 3:**
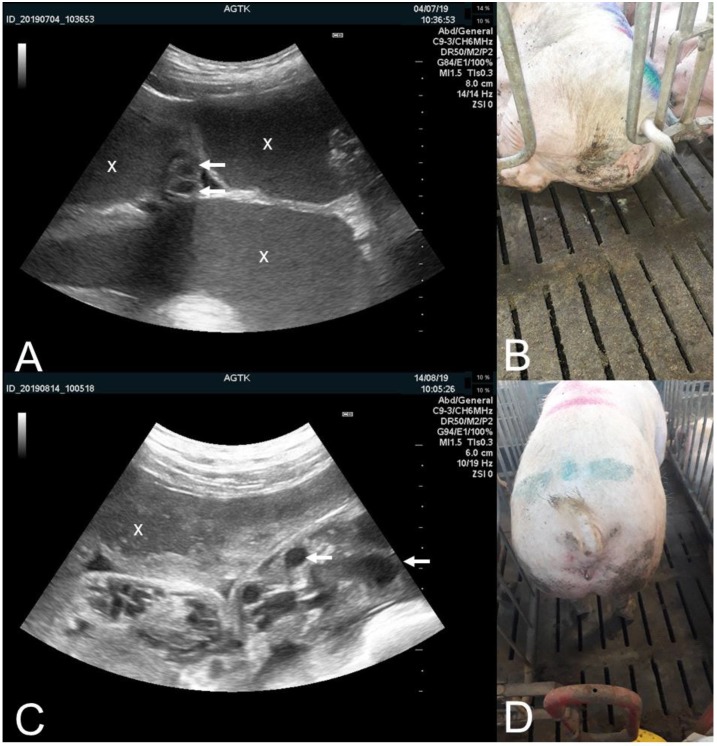
Transabdominal ultrasonographic images of uterine cross-sections (×) of sows assessed at (A) day of weaning (e.g., 28 days lactation) and (**C**) after a 14 days progesterone treatment initiated at weaning to postpone estrus. The uteri are filled with more (**C**) or less (**A**) flocculent fluid of moderate (**C**) or extreme volume (**A**). Uterine vessels are prominently enlarged (examples marked with arrows). (**B**) Moderate liquid purulent vulval discharge on the floor behind the sow passively drained off the sow (corresponds to image **A**). (**D**) Minor amounts of creamy purulent discharge in between vulval labia (corresponds to image **C**). (**A**,**C**) Scale bars on right margins in 0.5 cm steps.

The normal, non-gravid, post-puerperal (e.g., post-weaning) uterus is typically devoid of intrauterine fluid and, thus, should be absent of anechogenicity. Observation of any fluid echogenicity, beyond that observed within the first 12 h post-breeding, is indicative of a diseased uterus [1,2]. Most often, uterine fluid associated with a diseased condition does not present itself as a homogenous anechogenicity, but rather is more or less flocculent resembling “snow flurries” and suggestive of a severe acute endometritis (Figure 3) [1]. The amount of fluid present can vary considerably (Figure 3). Both, fluid appearance (more or less flocculent) and volume is associated with vulval discharge quality and consistency (Figure 3). Uterine vessels can also be observed to be enlarged in animals with severely inflamed uteri (Figure 3). In conjunction with these uterine findings, ovarian structures may be variable with CL (refer to Section 3.2. and Figure 7B) most often present, but observations of follicles of varying size may also be present. 

**Figure 4:**
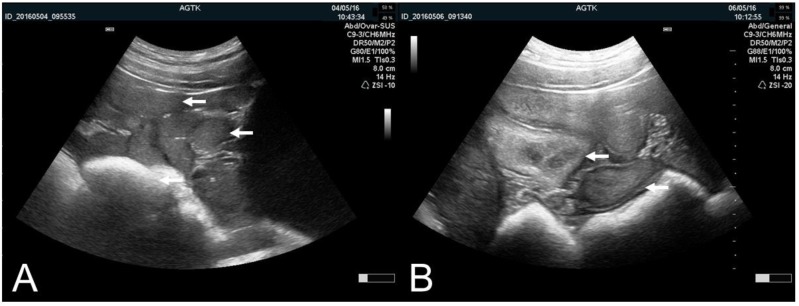
Transabdominal ultrasonographic images of uterine cross-sections (arrows) of sows in diestrus with a completely homogenous echotexture (**A**) and in estrus with a diffusely heterogeneous echotexture (**B**). Scale bar on right margin in 0.5 cm steps.

Uterine echotexture is another key parameter for the assessment of normalcy and functionality [1]. Uterine echotexture is steroid dependent and, thus, is primarily driven by ovary status [34]. In diestrous, when estrogens (E2) are low, the normal uterus appears completely homogeneous in its echotexture (Figure 4A) [1]. In all other stages of the estrous cycle, when E2 is typically above threshold levels, the uterus is heterogeneous in appearance (Figure 4B) [1]. Uterine exposure to E2 elicits an endometrial edema and hyperemia which leads to the aforementioned uterine heterogenicity. Consequently, as to whether a given uterine echotexture is physiological or not requires the simultaneous assessment of the ovarian status (i.e., as the source of steroids). A heterogeneous echotexture is associated with medium-to-large-sized follicles capable of producing sufficient amounts of E2. In diestrous, when CLs are present, progesterone is high and E2 low, the uterus is then expected to be devoid of any endometrial edema and hyperemia and, thus, should have a homogenous appearance. Other situations where E2 competent follicles are lacking (e.g., during lactation and at weaning) will also present as a homogenous, normal uterine echogenicity. Any divergence from this ovarian status-uterine echotexture relationship should be considered as suspicious for a diseased uterus [4]. In field investigations, if the uterus has heterogeneous echotexture in the presences of small follicles, subsequent sow fertility was reduced [35]. An extreme heterogeneous uterine echotexture in the presence of CLs in the sow has been associated with severe cases of endometritis in the absence of flocculent fluid [4]. A heterogeneous uterine echotexture in the presence of CLs has also been observed in field situations of sow mycotoxin (e.g., zearalenone) exposure [4]. 

The non-gravid post-puerperal uterus can also be assessed based on size. In terms of RTU, size is expressed as the cross-sectional area or the diameter of transversally cut uterine horns. Any uterine edema, whether physiological or pathological (see above), is associated with an increased uterine size [4,34]. Also, older parity sows typically have larger uterine sizes than younger animals [36]. Postmortem uterine weight has been used to determine uterine functionality [35]. A highly significant correlation between uterine weight and RTU measured size, best described by a polynomial regression equation, has been reported [4]. This equation was developed based upon data collected on non-gravid females (N=47) transcutaneously assessed at slaughter. Application of this equation to first service and repeat breeder females, however, failed to sufficiently predict animal fecundity [35]. 

Overall, the aforementioned supports added value in using RTU of the non-gravid post-puerperal uterus in the sow as a component of a diagnostic workup. This diagnostic modality, however, does have its limitations in relation to chronic endometritis in the sow. Repeated work has demonstrated that in cases of chronic endometritis, uterine appearance can be like that of a healthy uterus [1,5,37]. 

#### 3.1.4. The Pre-Pubertal Versus Pubertal Uterus

Transition from pre-pubertal to pubertal status in the gilt is associated with a phase of tremendous uterine growth, going from 100 g to 300 g [11]. Along with an increase in mass, a concomitant increase in uterine horn diameter is observed [11]. Assessment of uterine size via RTU, therefore, is the key parameter to assessing gilt uteri to determine pubertal status. The pre-pubertal uterus is visualized as a small structure that usually requires an added amount of abdominal probe pressure (when performed transabdominally) and time for proper visualization. The pubertal uterus, due to the fact of its larger size, is typically able to be visualized quite quickly after normal transabdominal probe placement and pressure [10,38]. Beyond this subjective determination of a gilt’s pubertal status, objective measurement of uterine size can be performed. In work done by Kauffold et al. [11], pre-pubertal gilts typically had a uterine horn cross-sectional area of ≤1.0 cm^2^, whereas pubertal gilts exhibited a cross-sectional area of ≥1.2 cm^2^. Animals which fall within the 0.2 cm^2^ gray zone are difficult to diagnose pubertal status based on uterine cross-sectional area. With animals falling within this “gray zone”, simultaneous imaging of the ovaries was required in order to achieve a diagnosis (refer to Section 4.1). Uterine horn diameter in gilts was not found to be a good predictor of uterine capacity [39]. It is important to note that gilts put on altrenogest may exhibit small amounts of anechoic intrauterine fluid, which should be considered normal without any apparent effects found on subsequent fertility [40].

A very recent study has employed Doppler sonography to assess uterine perfusion over the course of the estrous cycle in gilts [6]. In this study, non-anesthetized gilts were immobilized using a mobile purpose-designed crate. Using color-Doppler (e.g., not pulse-wave- and power-Doppler), the study reported a relationship between the level of perfusion and stage of the estrous cycle, with perfusion highest during the pro-estrus phase and lowest during metestrus and diestrus. 

### 3.2. The Ovary

**Figure 5:**
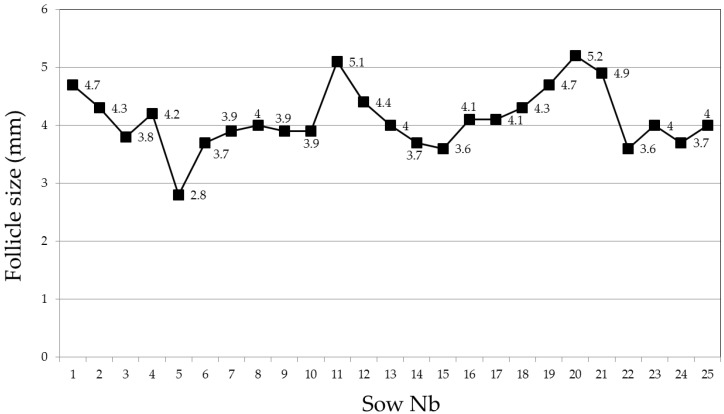
Follicle size of primiparous sows (n = 25) as determined by transabdominal ultrasonography one day post-weaning after 28 days of lactation [42].

**Figure 6:**
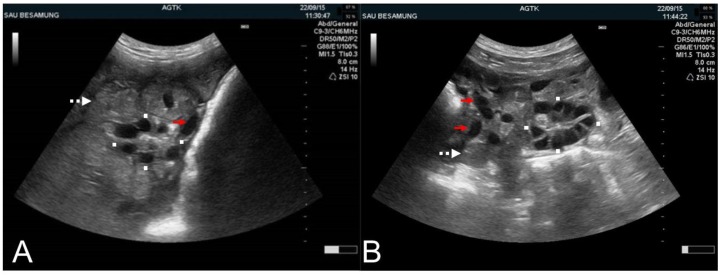
Transabdominal ultrasonographic images of ovaries in sows in estrus with round-oval (**A**) and polygonal pre-ovulatory follicles (**B**). (**A**,**B**) Squares mark the transversal and longitudinal dimension of the ovaries. Example cross-sections of the uterine horns (white, dotted arrows) and blood vessels (red arrows) are identified. Scale bars on right margins in 0.5 cm steps.

Ovaries and their structures can be visualized using high-resolution sonography in almost all relevant production stages in swine. Follicles are anechoic and can be identified at a 1 mm size [41]. Within one day of post-weaning (and incidentally at the end of an altrenogest treatment in gilts), follicles can be expected to measure between 3 to 5 mm in size (Figure 5) [42]. Pre-ovulatory follicles can vary in size ranging from approximately 6 mm [43] to 9 mm [44,45,46]. Follicle size does not seem to infer reproductive proclivity, as no association has been found to date between size and wean-to-estrus interval, fertilization rate, embryo development and diversity [46], farrowing rates or litter sizes [43]. As reviewed previously [1], pre-ovulatory follicles can exhibit a slight reduction in size and a change from a spherical to a more ovoid or longish shape [46,47,48] immediately preceding ovulation due to a loss in follicle turgidity [46]. Although these changes have been proposed to be candidate parameters to predict ovulation timing in pigs, this hypothesis has yet to be validated [1]. Recent work has provided some added insight in pre-ovulatory shape [49]. In this study, follicles of 10 multiparous sows were imaged and recorded at 6 hr intervals between 30 to 0 h prior to ovulation. Images were subsequently assessed for shape (round, oval and polygonal; Figure 6) using a computer software platform. Findings of this study found that as round shaped follicles decreased in number, a concomitant increase in numbers of polygonal shape structures occurred (Table 1). 

**Figure 7:**
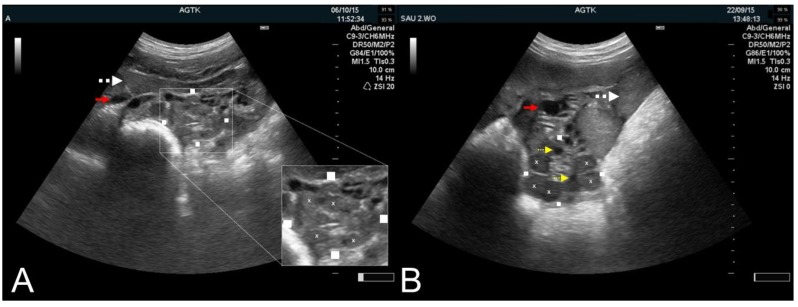
Transabdominal ultrasonographic images of ovaries in sows immediately post-ovulation (**A**) and in diestrus (**B**). Squares mark the transversal and longitudinal dimensions of the ovaries, and “×” corpora hemorrhagica (**A** inserted picture) or corpora lutea (**B**). Cross-sections of the uterine horns (white, dotted arrows), blood vessels (red arrows), and small follicles (yellow, dotted arrows) are identified. Note that in (**A**), there are no follicles visible. Scale bars on right margins in 0.5 cm steps.

Initiation of ovulation has also been determined through temporal RTU imaging of a sow’s ovaries in order to observe for a reduction in follicles suggestive of follicular rupture numbers [16,45]. Very shortly after follicle rupture (e.g., ovulation), the empty structure is filled with blood, leading to development of a corpus hemorrhagicum (CH). With better resolution RTU imagery, the CH can be clearly identified based on a characteristic echogenic appearance (Figure 7A) [8]. Observation of CHs is an excellent indicator for identifying sows who are peri- or post-ovulation [1]. Other indicators of ovulation, such as the inability to visualize the ovary due to the disappearance of follicles, have been reported [50]. These indirect methodologies for ovulation detection, however, have their limitations as in some circumstances the ovaries simply cannot be found due to the fact other tissue/structural obliteration (i.e., intestines, urinary bladder).

Frequent RTU examinations are required in order to accurately determine ovulation [1]. Examination ranges of 4 to 24 h have been reported when doing RTU for monitoring ovulation in pigs [45,46]. From an “on-farm” prospective, shorter intervals (e.g., 4 h) are not practical or necessary given that ovulation occurs over a short period of time [51]. Once a CH forms within the first few hours post-ovulation, it takes approximately 72 h to develop into a solid CL (Figure 7B). During this same time period, follicles development is frequently observed due to the FSH secretion in the sow which starts on as early as day 3 post-ovulation [52,53]. Early and mid-stage CLs appear similar in RTU imagery, making these structures of little value in determining the stage of diestrus in the estrous cycle. At approximately day 15 in the estrous cycle, CLs will start to regress, with RTU imagery showing a gradual reduction in CL diameter and size over the few days. A CL regresses to a corpus albicans (CA). A CA can only be imaged using high-resolution machines and is seen as a very small (i.e., 2–4 mm) hyperechoic node on the ovary.

Ovarian perfusion using color-Doppler ultrasonography has recently been assessed [54]. In contrast to the uterine findings [6], ovary perfusion was found to be highest in the diestrus phase of the estrous cycle. It was hypothesized that this observation may have been due, in part, to the fact that during other stages where follicular development was present, these ovarian regions were devoid of Doppler signals [54].

As with other species, ovarian cysts should be readily able to be imaged and diagnosed. Generally, single cysts have to be distinguished from the oligo-cystic (multiple cysts accompanied by CLs) and polycystic ovarian degeneration (POD) [1]. Cysts can be of different quality (i.e., follicular, luteal or “blood cysts”), but for prognosis, quantity is crucial. Due to the lack of treatment response, POD is usually an immediate reason for cull. If treatment is requested, altrenogest at 20 mg over 18 days has shown success in resolving follicular and luteal cysts [55]. Treatment with a GnRH-analogue may also be of value when treating for cystic structures in swine [56]. Delayed ovulation (i.e., the spread of ovulation over an interval >7 h) has been reported to occur, but is an uncommon herd problem [9,57]. Paraovarian cysts are frequently observed in swine but are not relevant to the animal’s fertility in the majority of situations [45,50,57]. Paraovarian cysts can vary in both their number and size (ranging from mm to multiple cm). Single or multiple paraovarian cysts are also possible. For differentiation from ovarian cysts, the location of the structures needs to be ascertained, with paraovarian cysts located topographically adjacent to an ovary.

## 4. Practical Use of RTU in Swine Reproduction

Due to the fact of its tremendous diagnostic capability, RTU has broad applicability for both “on farm” routine and troubleshooting work in female swine reproduction (Table 2) [13]. Examples will be briefly discussed herein.

### 4.1. Delayed or Failure to Attain Puberty

Timely attainment of puberty is important to gilt flow and breeding group management. It is critical that gilts be at a mature level by first breeding in order to facilitate higher production parameters throughout their breeding life (13]. In general, gilts culled for “no heat” should not exceed 5% in a production system. However, there is growing field evidence that delayed or failed puberty attainment is an increasing issue in some modern production systems. Diagnosis of reasons for pubertal failure is not always easy, as this problem is commonly multifactorial and includes factors related to management as well as to the environment (e.g., boar exposure, nutrition, health, and seasonality [13]. To complicate matters, a definitive diagnosis as to whether a puberty attainment problem is evident on a farm may also be influenced by changes in the herd’s genetic makeup, which have implications on age/weight till maturity. Estrus detection via behavioral and visual signs can be ambiguous, as peri-pubertal gilts may display signs of estrus such as reddening and swelling of the vulva, but fail to elicit a strong standing reflex. Indeed, some of these animals will fail to ovulate, while others will. Given the aforementioned, application of RTU becomes the only way to accurately determine the puberty status in the live animal if performed according to the aforementioned criteria (refer to Section 3.1.4.). According to Kauffold et al. [11], optimal diagnosis accuracy is achieved if both the uterus and the ovaries are examined. If it is assumed that puberty occurs in a line of gilts at 180 days of age, a workup to determine the pubertal status in that system may include three or more age groups starting at around 180 days, with further groups bracketed in 20 day increments. In the same system, older gilts with reported “no-heat” status should also be RTU examined. This proposed approach will allow the clinician to better understand the problems’ magnitude and to exclude poor estrus detection management from the list of differentials [13]. Real-time ultrasonography imaging for the gilt’s pubertal status may also be helpful prior to implementing use of exogenous hormones for puberty induction in a system in order to ensure appropriate pre-pubertal status at treatment [37]. In cases where exogenous hormonal treatment (e.g., altrenogest) response is less than desired, RTU can be helpful in ascertaining whether the poorly responding gilts may have been improperly treated (i.e., too low or high a dosage, improper treatment application) or may not be pubertal eligible at the time treatment was started [58]. Ovary imaging using RTU at the beginning and end of treatment can greatly aid in diagnosing and correcting the problem.

### 4.2. Prolonged Wean-to-Estrus Interval

Ideally, weaned sows should exhibit estrus within 7 days post-weaning. If estrus is not observed, then animals may be considered either “missed” or “truly failed” sows [13]. Reasons for “missed heats” are either silent estrus or, in the majority of cases, improper estrus detection that is clearly management related. If a sow “truly fails to be in heat”, the reason may be ovarian inactivity or lactational estrus [59,60], both of which can be influenced by the weaning age. In either case, both are predominantly metabolically driven, with the former being associated with an animal in catabolic status while the latter one in anabolic status, especially later into lactation [61,62]. Other intrinsic and extrinsic factors may play a role, as ovarian inactivity can be parity-associated (commonly known as “second-litter syndrome” in primiparous sows) or due to insufficient boar stimulation post-weaning. Lactational estrus may be caused by seasonality/low ambient temperature (typically seen in late autumn and winter in northern hemisphere), or influenced by lactation length (e.g., systems with ≥ 4 weeks of lactation), or in systems which employ practices which interrupt suckling continuity. In these situations, the clinical presentation (i.e., not showing estrus when expected) typically does not allow for a definitive diagnosis as to the etiology and pathogenesis. Since post-weaning estrus is ultimately ovary dependent, RTU of the ovaries of suspected sows can greatly aid in understanding the problem and, thus, prescribe appropriate treatment and/or prevention strategies. Imaging of suspected sows around day 7 post-weaning is a good starting point in a diagnostic workup [13]. At this time post-weaning, missed heat sows will likely have CH, sows with ovarian inactivity only small follicles, and sows having lactational estrus showing CLs on their ovaries. Note that overlap may occur with lactational estrus and “missed” early ovulating sows, as both may have functional CLs present. If lactational estrus is suspected, RTU imaging of sows on the day of weaning will provide the necessary evidence to make the diagnosis definitive. As a side note, field observations have observed POD on a few sows, believed to have been induced as a result of follicular growth during lactation lacking appropriate hormonal stimulus for ovulation to occur [63].

### 4.3. Problems with Conception and Farrowing Rate

Disruptions in conception and farrowing rate may be a combination of infectious and non-infectious causes and may extend to both the female and male components of breeding [13]. In today’s production systems, it is not unreasonable to expect conception rates to exceed 90%, with at least an 88% farrowing rate. If problems are occurring, an appreciation of normal reproductive physiology during conception and pregnancy is paramount to a successful diagnostic approach. Once gametes fuse and an embryo is formed, embryos must migrate throughout the uterus before implanting, embryonic membranes develop, and fetal growth and maintenance of pregnancy ensue through term. In all of these steps, a healthy animal and their genital tract is required. In addition, functional endocrinological pathways must be in place to allow for ovulation, maternal recognition of pregnancy, and pregnancy maintenance vis-a-vis the presence of functional CLs. Numerous extrinsic factors may interfere with the establishment and maintenance of pregnancy, including nutrition, seasonality/ambient temperature, housing (including day post breeding when sows are moved from breeding into gestation), exogenous hormonal use, etc. [13]. Generally, if decreases in conception and farrowing rates are observed, a prerequisite to an on-site visit would be to perform a thorough data and protocol analysis of that farm in order to prioritize diagnostic measures, including where/when to apply RTU. In this data review, parity status is a crucial blocking factor as certain early parity sows can be more susceptible to nutritional influences or, according to field observation, may reproductively behave differently than older sows in systems where exogenous hormones are being used [64]. When a sow returns to estrus is of particular value in a diagnostic workup. With a regular return to estrus, defined as animals showing estrus 18–24 days post breeding, these types of returns are usually driven by non-infectious (management-related) causes with inappropriate breeding practices being a primary driver. Irregular returns to estrus lend themselves to being caused by infectious pathogens as the primary reason [13]. In either situation, starting with an RTU assessment of ovarian dynamics in association with breeding management is a good initial diagnostic step with the goal of including (regular returns) or excluding (irregular returns) improper breeding times as the reason for failed conception. The next step would then be to RTU assess (very) early pregnancy checks starting around day 16 post breeding (earlier, depending upon equipment; refer to Section 3.1.1.) in order to include/exclude embryonic mortality as the cause. Confirmed open females should also be imaged to check for ovarian/uterine disease, with suspect animals tagged for euthanasia and necropsy.

A similar step in the diagnostic workup is done when addressing low farrowing rates or so-called increased late fallouts (i.e., animals checked pregnancy positive but fail to farrow). The aforementioned approach for disrupted conception rates is performed in order to determine if breeding errors are a root cause of low farrowing rates. Repeated assessment of pregnancy status using RTU in one or more breeding groups starting at the farm’s typical week of pregnancy testing (i.e., according to the farm’s standard operation procedure), and then repeated weekly over the entire period of pregnancy may be required in order to delineate the extent (e.g., magnitude and time of insult) of the problem. These findings would drive precision timing of downstream diagnostics such as serology or management related issues (e.g., vaccination timing, loose sow grouping, animal handling, etc.). Lastly, farrowing rate problems (and subsequently late fallout problems) can also be the result from errors in a farm’s pregnancy testing program. If this is suspected, it is strongly advised to initiate the diagnostic workup starting with the estrus detection screening process along with assessing the capabilities and accuracy of any on-site personnel performing ultrasonography pregnancy confirmation [13].

### 4.4. Problems with Non-Puerperal Vulval Discharge

Vulval discharge is considered a normal component when it occurs ante- and post-farrowing and while an animal is in estrus. Additionally, it is typical for sows to show a vulval discharge shortly after artificial insemination, with no detrimental effects on fertility. In any other situation, vulval discharge is an indicator of an underlying disease process in the reproductive tract [37]. Discharges may originate from the genital or the urinary tract. Single discharging animals are common, but if continuously observed in a frequency ≥ 3%, it should be classified as problematic and be investigated [13]. Quality and quantity of discharge may be suggestive but is not definitive to origin. Discharge from the urinary tract can have mucus present in it and is usually observed when expelled together with last stream of urine. Discharges originating from the genital tract are more prone to being milky or creamy and purulent, and can be seen being discharged while the animal is recumbent (refer to Section 3.1.3 and Figure 3). All parts of the genital tract can elicit a discharge if inflamed. However, vulval discharges are more frequently associated with uterine inflammation rather than of cervical or vaginal origin [37]. Inflammation can be caused by different pathogenic organisms. While under normal circumstances the uterus is sterile, bacterial entry may occur at parturition (e.g., obstetrical interventions) or breeding (e.g., contaminated semen, dirty insemination procedure) when the cervix is open. Chlamydial species, however, may be able to colonize the genital tract through blood dissemination. As to whether a bacterial colonialization is cleared or not certainly depends on the bacterial type and load [37].

Other factors can contribute to genital inflammation. For instance, post-ovulatory inseminations performed at times under progesterone (P4) predominance may facilitate inflammation, as P4 is immunosuppressive in the genital tract. A similar immunosuppressive effect can be caused in animals exposed to feed contamination containing the mycotoxin deoxynivalenol. A puerperal endometritis that is left under- or non-treated can become chronic as the animal enters into the breeding pool, allowing the re-emergence of vulval discharge upon re-entry of pathogens at breeding. Commonly, affected animals start discharging any time from day 10 post-breeding, but earlier discharges are possible. RTU imagery can aid in defining the discharge’s origin (refer to Section 3.1.3.) when assessing an animal with clinical signs. Lastly, RTU imaging of ovarian structures may be of value when assessing females exhibiting a vulval discharge. Value in this application was found on a farm employing a program for estrus synchronization and ovulation with fixed time insemination. When assessing ovarian structures, approximately 4% of the sows were being bred while having a functional CL present. These sows, subsequently, developed a vulval discharge.

## 5. Conclusions

Since its initial application in the 1980s, RTU has rapidly gained favor in its diagnostic value related to swine reproduction. With RTU, virtually all tissues of the female genital tract can be accurately assessed in all stages of production (e.g., gilt development, breeding, gestation and farrowing). Though RTU is most commonly used on-farm for assessing pregnancy status, it has become an essential diagnostic tool when troubleshooting disruptions to herd reproductive performance. Particular issues where RTU has repeatedly provided diagnostic value include delayed puberty, prolonged wean-to-estrus interval, absence of post-weaning estrus, herd disruptions in conception and farrowing rates, vulval discharge, peripartum and puerperal disorders. If combined with doppler, RTU can provide added value not just in fetal viability assessment, but also in assessing perfusion characteristics of the uterus and the ovaries that may aid in achieving an appropriate diagnosis.

## Figures and Tables

**Table 1:** Breakdown of follicle shape in relation to time prior to ovulation in multiparous sows (n = 10) imaged transabdominally at 6 h intervals [modified according to 49].

Interval Prior to Ovulation (h)	Follicles Analyzed (*n*)	Follicles “Round” (%)	Follicles “Oval” (%)	Follicles “Polygonal” (%)
30–24	94	26.6	26.6	46.8
24–18	95	13.7	34.7	51.6
18–12	103	10.7	27.2	62.1
18–6	42	4.8	38.1	57.1
6–0	47	0	27.7	72.3

**Table 2:** Applicability of ultrasonography in female swine reproduction and for troubleshooting of “on farm” fertility problems [13].

Applicability in Female Swine Reproduction ^1^	Applicability for Troubleshooting of “on Farm” Fertility Problems ^1^
Diagnosis of pregnancy and pregnancy failure Assessment of health of the non-pregnant uterus Monitoring of ovulation and ovulation failure Assessment of puberty and failure to attain puberty Assessment of follicle growth and failure to grow Determination of fetal viability, retained piglets and placentae, assessment of uterine involution (Measurement of backfat depth)	Low conception rate Low farrowing rate Late fallouts High rate of returns Delayed puberty/high cull rate of gilts for “No Heat“ Vulval discharges Reduced litter size Long wean–estrus interval Peripartum/puerperal uterine disorders (Body condition monitoring)

^1^ Fertility-associated applications are in parentheses.
